# Visit-to-visit blood pressure variability and the risk of stroke in the Netherlands: A population-based cohort study

**DOI:** 10.1371/journal.pmed.1003942

**Published:** 2022-03-17

**Authors:** Alis Heshmatollah, Yuan Ma, Lana Fani, Peter J. Koudstaal, M. Arfan Ikram, M. Kamran Ikram

**Affiliations:** 1 Department of Epidemiology, Erasmus MC University Medical Center, Rotterdam, the Netherlands; 2 Department of Neurology, Erasmus MC University Medical Center, Rotterdam, the Netherlands; 3 Department of Epidemiology, Harvard T.H. Chan School of Public Health, Boston, Massachusetts, United States of America; Columbia University, UNITED STATES

## Abstract

**Background:**

Apart from blood pressure level itself, variation in blood pressure has been implicated in the development of stroke in subgroups at high cardiovascular risk. We determined the association between visit-to-visit blood pressure variability and stroke risk in the general population, taking into account the size and direction of variation and several time intervals prior to stroke diagnosis.

**Methods and findings:**

From 1990 to 2016, we included 9,958 stroke-free participants of the population-based Rotterdam Study in the Netherlands. This is a prospective cohort study including participants aged 45 years and older. Systolic blood pressure (SBP) variability was calculated as absolute SBP difference divided by mean SBP over 2 sequential visits (median 4.6 years apart). Directional SBP variability was defined as SBP difference over 2 visits divided by mean SBP. Using time-varying Cox proportional hazards models adjusted for age, sex, mean SBP, and cardiovascular risk factors, hazard ratios (HRs) for stroke up to January 2016 were estimated per SD increase and in tertiles of variability. We also conducted analyses with 3-, 6-, and 9-year intervals between variability measurement and stroke assessment. These analyses were repeated for diastolic blood pressure (DBP). The mean age of the study population was 67.4 ± 8.2 years and 5,776 (58.0%) were women. During a median follow-up of 10.1 years, 971 (9.8%) participants had a stroke, including 641 ischemic, 89 hemorrhagic, and 241 unspecified strokes. SBP variability was associated with an increased risk of hemorrhagic stroke (HR per SD 1.27, 95% CI 1.05–1.54, *p* = 0.02) and unspecified stroke (HR per SD 1.21, 95% CI 1.09–1.34, *p <* 0.001). The associations were stronger for all stroke subtypes with longer time intervals; the HR for any stroke was 1.29 (95% CI 1.21–1.36, *p <* 0.001) at 3 years, 1.47 (95% CI 1.35–1.59, *p <* 0.001) at 6 years, and 1.38 (95%CI 1.24–1.51, *p <* 0.001) at 9 years. For DBP variability, we found an association with unspecified stroke risk. Both the rise and fall of SBP and the fall of DBP were associated with an increased risk for unspecified stroke. Limitations of the study include that, due to an average interval of 4 years between visits, our findings may not be generalizable to blood pressure variability over shorter periods.

**Conclusions:**

In this population-based study, we found that visit-to-visit blood pressure variation was associated with an increased risk of unspecified and hemorrhagic stroke, independent of direction of variation or mean blood pressure.

## Abstract 2

In a population-based cohort study, Alis Heshmatollah and colleagues investigate the associations between blood pressure variability and risk of stroke among adults in the Netherlands.

## Author summary

**Why was this study done?:**

- Increased blood pressure is a known and major risk factor of stroke. Growing evidence suggests that, despite hypertension control and medication adherence, visit-to-visit variation in blood pressure is also a risk factor for stroke in high-risk populations.
- It is not known whether variation in blood pressure is also associated with an increased risk of stroke in the general population.

**What did the researchers do and find?:**

- In this analysis of a prospective cohort study, we followed 9,958 stroke-free participants between 1990 and 2016 and measured blood pressure variation over 2 sequential visits that were an average of 4 years apart.
- We found that variation of systolic blood pressure, independent of its direction, was associated with an increased risk of stroke, in particular for hemorrhagic and unspecified stroke.
- Regarding diastolic blood pressure, we found that variation, and in particular a fall but not a rise, in diastolic blood pressure was associated with unspecified stroke.

**What do these findings mean?:**

- Blood pressure variability may be a modifiable risk factor for stroke, and future studies should focus on whether lowering blood pressure variability results in lower stroke incidence.

## Introduction

Hypertension is a major modifiable risk factor for stroke and cardiovascular events [1]. From more recent research, visit-to-visit variation in blood pressure is thought to play a role in the etiology of stroke, in addition to blood pressure level alone. A major hypothesis is that a larger variation in blood pressure increases mechanical stress on the vascular system, accelerating several pathophysiological mechanisms including atherosclerosis [2,3]. Indeed, growing evidence suggests that, despite hypertension control and medication adherence, visit-to-visit variation in blood pressure is a risk factor for stroke, cardiovascular disease, and mortality [2,4–6].

However, previous studies on the association between blood pressure variation and stroke have primarily been conducted within trials of high-risk individuals with hypertension, high cardiovascular risk, or previous stroke or transient ischemic attack, or within post-menopausal women [6–10]. Population-based studies on this topic are scarce, have also focused on specific subgroups, and have yielded heterogeneous results [11–13]. Blood pressure variability was found to be strongly associated with cardiovascular events including stroke in chronic kidney disease patients [11], yet other studies among participants with hypertension or antihypertensive medication use did not confirm the link between blood pressure variability and stroke risk [12,13]. In addition, when focusing on stroke subtypes, the evidence is limited and conflicting [7,10,13].

Thus, studies within the general population are needed to determine the clinical relevance of blood pressure variability in the risk of stroke and its subtypes. Moreover, such studies can provide further evidence for future interventional trials that focus on reducing blood pressure variability in addition to blood pressure level.

In this analysis of a prospective population-based study, we determined the association between visit-to-visit blood pressure variability and the risk of stroke and its subtypes, taking into account the magnitude and direction of variability and several time intervals prior to stroke diagnosis.

## Methods

### Study setting and population

This study is based on the Rotterdam Study, a large prospective population-based cohort in the Netherlands designed to study the occurrence and determinants of diseases in the general population [14]. The cohort was initiated in 1990 (RS-I) with 7,983 participants and was expanded by 3,011 participants in 2000 (RS-II) and by 3,932 participants in 2006 (RS-III). In total, 14,926 participants aged 45 years or older have participated in the study. Examination rounds with home interviews and extensive physical examinations are repeated on average every 4 years. Of the 14,926 participants, 12,120 attended the first and second examination round of their cohort. Participants were excluded if they had missing blood pressure measurements at the first 2 examinations (*n =* 1,732), no informed consent for follow-up data collection (*n =* 74), previous stroke at first examination (*n =* 239), or incident stroke between the first and second examination (*n =* 103). In total, 9,958 participants were left for the systolic blood pressure analysis. For the analysis of diastolic blood pressure variability, an additional 3 participants with missing diastolic blood pressure measurements were excluded, resulting in 9,955 individuals for analysis.

The Rotterdam Study was approved by the Medical Ethics Committee of Erasmus MC (registration number MEC 02.1015) and by the Dutch Ministry of Health, Welfare and Sport (Population Screening Act WBO, license number 1071272-159521-PG). All participants provided written informed consent to participate in the study. This study is reported as per the Strengthening the Reporting of Observational Studies in Epidemiology (STROBE) guideline (S1 Text).

### Blood pressure variability

**Fig 1:**
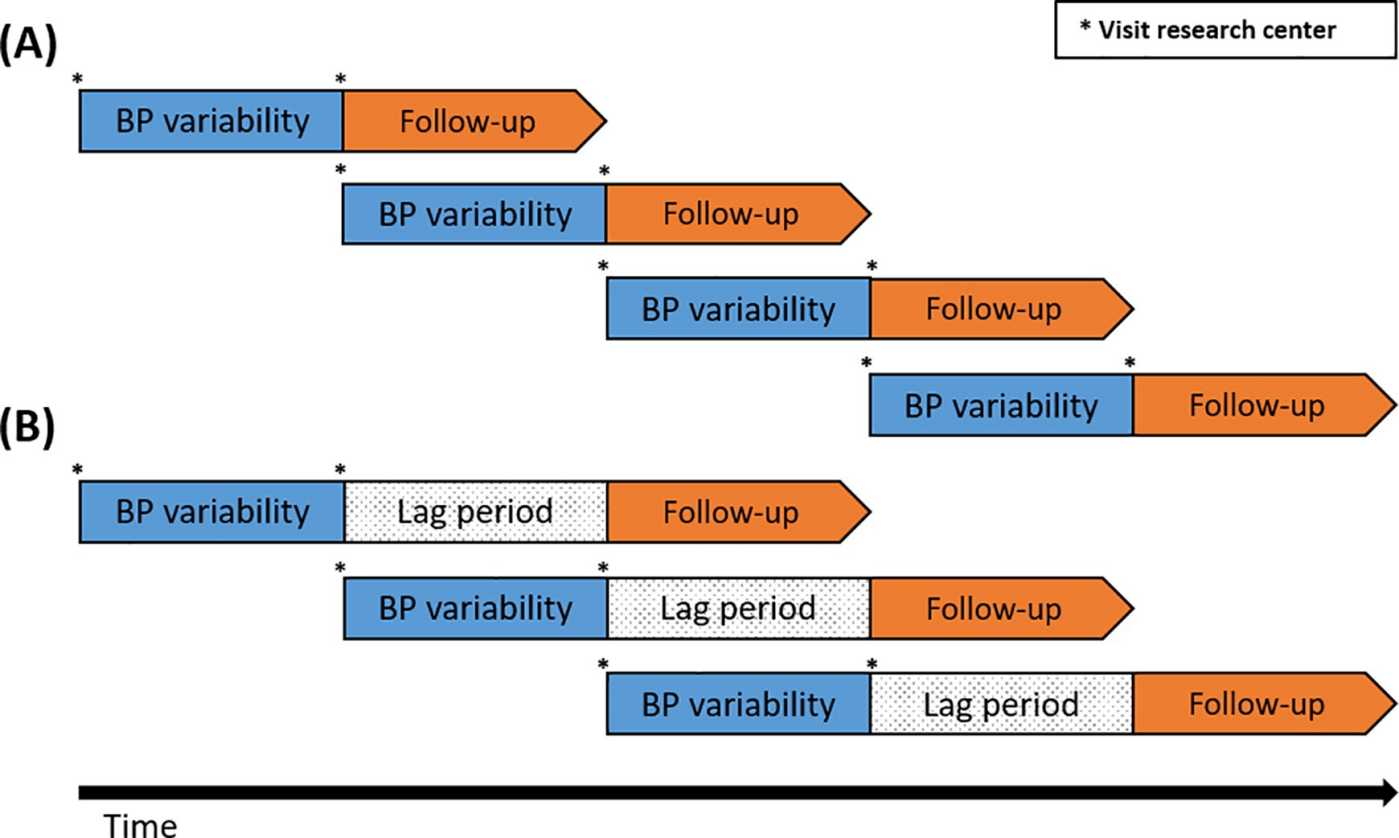
Schematic presentation of the analyses with and without a lag period. (A) Analyses without lag periods, in which blood pressure (BP) variability is measured over 2 visits with immediate follow-up for stroke incidence thereafter. Blood pressure variability is then updated when the participant visits the research center again. (B) Analyses with lag periods. After blood pressure variability measurement, a lag period of 3, 6, or 9 years is incorporated before follow-up for incident stroke to account for reversed causation.

During each examination at the research center, blood pressure was measured twice at the right upper arm after at least 5 minutes of rest in a seated position. The mean of the 2 readings was used. Blood pressure was measured in the same way in all examination rounds. Before 7 November 2006, a Hawksley random-zero sphygmomanometer was used. Omron M6 Comfort and Omron M7 devices were used thereafter. Systolic blood pressure variability within individuals between 2 sequential visits was defined as the absolute difference in systolic blood pressure divided by the mean systolic blood pressure over the 2 visits (|difference|/mean) and then transformed into a normal standardized distribution. This formula is easily translated into clinical practice and also allows calculation of the percentage difference: (|difference|/mean) × 100%. Furthermore, the formula can be adapted to calculate directional systolic blood pressure variability, which differentiates rises and falls in systolic blood pressure. This was defined as the difference in systolic blood pressure between the 2 visits divided by the mean ([latter − former]/mean). We recently used this same approach in other studies assessing variation in blood pressure [15,16]. To account for different examination intervals (median 4.6 years, range 1.9 to 6.5 years), both measurements were scaled to the average variability per year, assuming a constant rate of variability between the 2 visits. The same formulas were used to calculate measurements of diastolic blood pressure variability. Blood pressure variability was assessed as a time-varying exposure. For the first blood pressure variability measurement, the first and second examination were used; after the third examination round, blood pressure variability was updated using the second and third examinations, and so on (Fig 1A). If a participant missed an examination round (*n =* 145), blood pressure variability assessed at the previous examination was used.

### Stroke ascertainment

Stroke was defined according to the World Health Organization (WHO) criteria as a syndrome of rapidly developing clinical signs of focal or global disturbance of cerebral function, with symptoms lasting 24 hours or longer or leading to death, with no apparent cause other than of vascular origin [17]. History of stroke at study entry was assessed during the baseline interview and verified by medical records. After enrollment, participants were continuously monitored for incident stroke through automated linkage of the study database with files from general practitioners and nursing home physicians. Files from nursing home physicians and general practitioners of participants who moved out of the district were also checked on a regular basis. Additional information was obtained from hospital records. The information collected on potential strokes was reviewed and classified by research physicians and verified in a consensus panel led by 2 experienced stroke neurologists. Final stroke diagnosis was adjudicated in accordance with the abovementioned standardized diagnostic WHO criteria, which were held constant over the entire follow-up time. Strokes were further classified as ischemic or primary intracerebral hemorrhage based on neuroimaging reports or hospital discharge letters. If neuroimaging was not conducted or reported, a stroke was classified as unspecified. This classification into ischemic, hemorrhagic and unspecified stroke corresponds with ICD-10 codes I61, I63, and I64. In the stroke analysis, participants could contribute to follow-up until first-ever stroke, death, loss to follow-up, or last health status update when they were known to be free of stroke, whichever came first, until January 1st 2016. Follow-up was virtually complete (97.8%).

### Other measurements

Participants were interviewed at home every 4 to 5 years with standardized questionnaires on health status, medical history, and medication use including use of blood-pressure-lowering medication, lipid-lowering medication, and anti-thrombotic agents. They additionally underwent physical examinations on average every 4 years at the dedicated research center. Participant educational level was categorized as primary education, lower education, intermediate vocational or higher general education, and higher vocational education or university. Smoking status was classified as current, former, or never. Alcohol consumption was recorded as grams of alcohol intake per week. Body mass index (BMI) was calculated as weight divided by the square of height (kg/m^2^). Blood samples were drawn to assess levels of cholesterol, high-density lipoprotein cholesterol (HDL cholesterol), and glucose. Type 2 diabetes mellitus was defined as a fasting glucose of ≥7.0 mmol/L, a non-fasting or post-load serum glucose of ≥11.1 mmol/L, or use of blood-glucose-lowering medication. Additionally, diagnosis of type 2 diabetes mellitus, atrial fibrillation, and coronary heart disease was based on repeated screening and review of medical records.

### Statistical analysis

Missing values on covariates were imputed and pooled using 5-fold imputation. The percentage of values missing ranged from 1.9% to 9.7%, except for diabetes mellitus, for which 18.7% of the values were missing.

Cox proportional hazards models were used to estimate hazard ratios (HRs) for the association between systolic and diastolic blood pressure variability measurements and the risk of incident any stroke, ischemic stroke, hemorrhagic stroke, and unspecified stroke. Blood pressure variability was assessed both continuously and after categorizing it into tertiles, with the reference group defined as the lowest tertile for absolute variability. Directional blood pressure variability was also divided into tertiles, but using the middle tertile as the reference group. We used time-independent (sex and education) and time-dependent covariates (age, mean systolic or diastolic blood pressure, blood-pressure-lowering medication use, BMI, alcohol consumption, smoking, total cholesterol, HDL cholesterol, lipid-lowering medication use, anti-thrombotic medication use, type 2 diabetes mellitus, atrial fibrillation, prior coronary heart disease, and type of blood pressure measuring device), which were updated simultaneously with blood pressure variability after each visit to the research center. The proportional hazards assumption was met. This assumption was tested by assessing the relationship between the scaled Schoenfeld residuals of each covariate and time, and by additional graphical inspection of these residuals against time for any non-random pattern. Model I was adjusted for age, sex, and mean systolic or diastolic blood pressure (whichever was applicable), and model II additionally included all other abovementioned covariates. We then repeated the analyses with varied latency periods to account for possible reverse causation. We estimated the associations considering a lag period of 3, 6, and 9 years (Fig 1). The years of lag represent the minimum interval between the systolic blood pressure variability measurements and the assessment of incident stroke. For example, when the lag period was 0, we investigated incident stroke cases immediately after the blood pressure variability measurement, whereas with a lag of 3 years we investigated stroke cases at least 3 years after the blood pressure variability measurement. Using this method, participants with less than 3 years of follow-up could not be included in this analysis, those with less than 6 years of follow-up were excluded from the lag 6 analysis, and so on. To minimize selection bias in these lag analyses, we used inverse probability weights in the Cox proportional hazards models [18–20]. These weights represent the inverse probability of being included in the period-specific analysis out of the overall study population. The numerator of these weights modeled the probability of being included without considering covariates, and the denominator modeled the probability of being included considering all the covariates that were also used in the primary analysis. The weights were truncated at the first and 99th percentiles, and Robust sandwich estimators were used to allow for the dependence of weighted participants. To avoid overfitting in these lag-specific analyses with smaller numbers of participants, we calculated generalized propensity scores for blood pressure variability and directional blood pressure variability based on all covariates except age, sex, and mean systolic or diastolic blood pressure [21]. This propensity score was then added as a covariate to the Cox model along with age, sex, and mean systolic or diastolic blood pressure.

Furthermore, to assess potential effect modification, we stratified the analysis of systolic blood pressure variability and the risk of any stroke by age (median as cut point), sex, systolic blood pressure (mean as cut point), and use of blood-pressure-lowering medication. We adjusted these analyses for age, sex, mean systolic blood pressure, and the previously mentioned propensity score. For the stratification by blood-pressure-lowering medication use, we calculated a new generalized propensity score that did not include blood pressure medication use.

There was no documented prospective analysis plan for the study. However, analyses were planned before examination of the data, and we made no data-driven changes to the analyses. In response to peer review, we added alcohol consumption, anti-thrombotic medication use, and type of blood pressure measuring device as adjustments in model II. We additionally tested whether there was an interaction between systolic blood pressure variability and stroke and its subtypes by adding an interaction term in the model (systolic blood pressure variability × baseline systolic blood pressure). Finally, we repeated the systolic blood pressure variation analysis using only complete cases.

Alpha level (type I error) was set at 0.05. All statistical analyses were performed using R 4.1.1 software.

## Results

Of the 9,958 participants included, 5,776 (58.0%) were women, and mean age was 67.4 ± 8.2 years (Table 1). At baseline, mean systolic blood pressure was 139 ± 19 mm Hg, and mean diastolic blood pressure was 78 ± 10 mm Hg. During a median follow-up of 10.1 years (interquartile range 2.8 to 15.4), 971 (9.8%) participants experienced an incident stroke, of whom 641 (66.0%) had ischemic stroke, 89 (9.2%) had hemorrhagic stroke, and 241 (24.8%) had unspecified stroke.

**Table 1:** Baseline characteristics of the study population.

Characteristic	Study population, *n* = 9,958
Age, years	67.4 ± 8.2
Women	5,776 (58.0%)
Educational level	
Primary	1,483 (14.9%)
Lower	4,039 (40.6%)
Intermediate	2,837 (28.5%)
Higher	1,600 (16.1%)
Systolic blood pressure, mm Hg	139 ± 19
Diastolic blood pressure, mm Hg	78 ± 10
Blood-pressure-lowering medication use	3,059 (30.7%)
Body mass index, kg/m^2^	27.0 ± 4.1
Alcohol consumption, g/day	6.1 ± 9.3
Smoking status	
Never	3,018 (30.3%)
Former	3,378 (33.9%)
Current	3,562 (35.8%)
Total cholesterol, mmol/L	6.2 ± 1.3
High-density lipoprotein cholesterol, mmol/L	1.4 ± 0.4
Lipid-lowering medication use	1,307 (13.1%)
Anti-thrombotic medication use	1,017 (10.2%)
Type 2 diabetes mellitus	1,347 (13.5%)
Atrial fibrillation	473 (4.7%)
Prior coronary heart disease	717 (7.2%)

Data presented as frequency (percentage) for categorical values and mean ± standard deviation for continuous variables.

### Blood pressure variability

**Fig 2:**
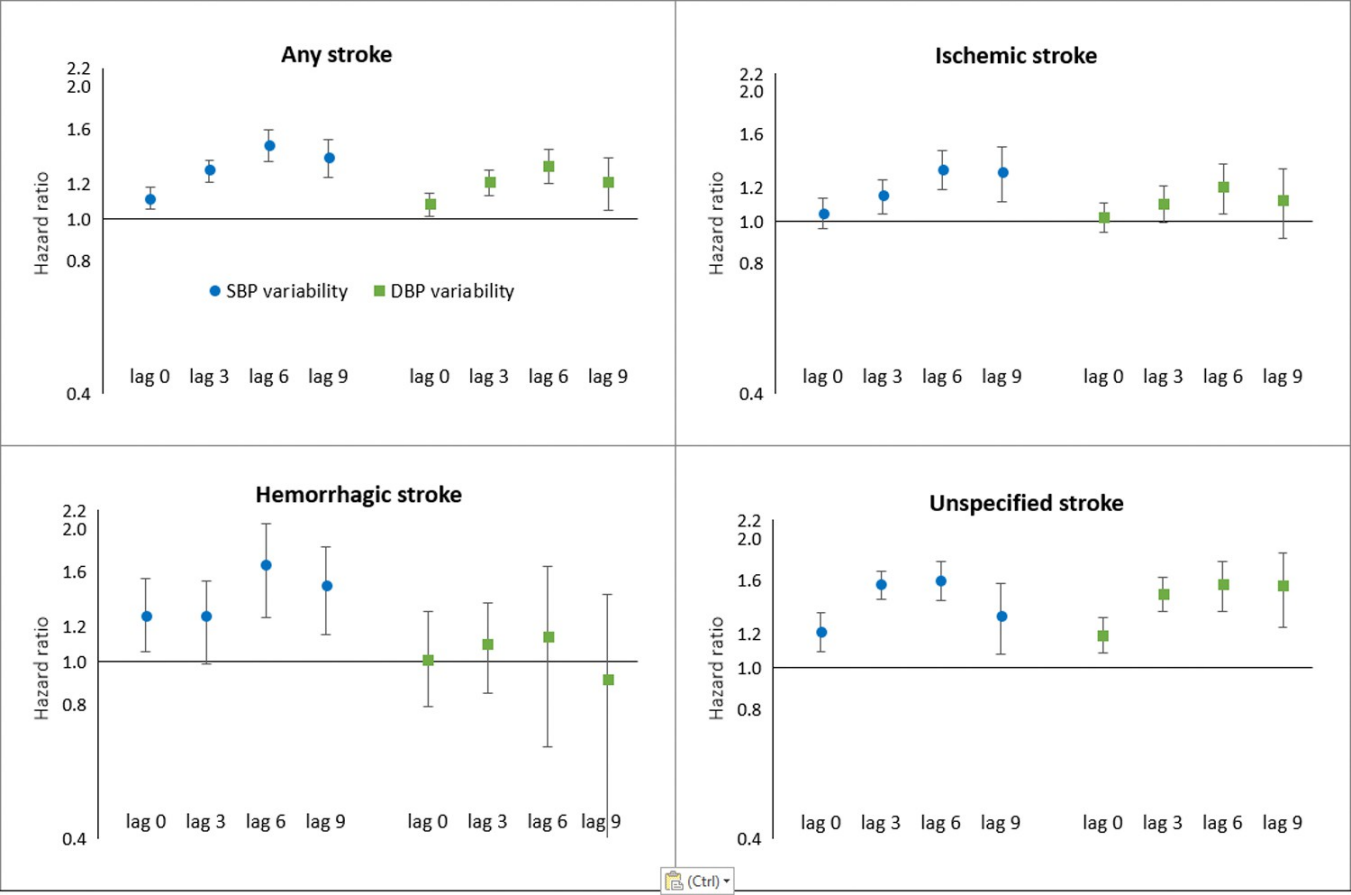
The association between blood pressure variability and incident stroke using different lag periods. The blue dots and green squares represent the hazard ratio of incident stroke per SD increase in systolic blood pressure (SBP) variability and diastolic blood pressure (DBP) variability, respectively. The error bars show the 95% confidence intervals around the estimates. The lag 0 models were adjusted for age, sex, mean SBP or DBP, education, BMI, smoking, alcohol consumption, cholesterol, high-density lipoprotein cholesterol, lipid-lowering medication use, blood-pressure-lowering medication use, anti-thrombotic medication use, type 2 diabetes mellitus, atrial fibrillation, prior coronary heart disease, and type of blood pressure measuring device. Lag 3, lag 6, and lag 9 analyses were adjusted for age, sex, mean SBP or DBP, and propensity score based on all other covariates. Lag 0 represents no time interval between blood pressure variability measurement and follow-up for incident stroke, lag 3 represents a time interval of 3 years between blood pressure variability measurement and follow-up for incident stroke, and so on.

Systolic blood pressure variability was associated with any stroke (HR per SD increase 1.11, 95% CI 1.05–1.18, *p <* 0.001), hemorrhagic stroke (HR 1.27, 95% CI 1.05–1.54, *p =* 0.02), and unspecified stroke (HR 1.21, 95% CI 1.09–1.34, *p <* 0.001) in model II, adjusted for all covariates (Table 2). The association with ischemic stroke did not reach statistical significance (HR 1.04, 95% CI 0.96–1.13, *p =* 0.35). Diastolic blood pressure variability was associated with any stroke (HR 1.08, 95% CI 1.01–1.14, *p =* 0.02) and unspecified stroke (HR 1.19, 95% CI 1.08–1.31, *p <* 0.001) only. When exploring tertiles of blood pressure variability, we found that especially the highest tertile of variability was associated with an increased risk of unspecified stroke for both systolic (HR 1.64, 95% CI 1.16–2.31, *p <* 0.001) and diastolic blood pressure (HR 1.43, 95% CI 1.03–1.99, *p =* 0.03). Furthermore, the lag-period-specific analyses revealed that the association between systolic blood pressure variability and incident stroke and all stroke subtypes was stronger over longer intervals up to a lag period of 6 years (HR for any stroke [lag 3] 1.29, 95% CI 1.21–1.36, *p <* 0.001; HR [lag 6] 1.47, 95% CI 1.35–1.59, *p <* 0.001; HR [lag 9] 1.38, 95% CI 1.24–1.51, *p <* 0.001) (Table 3; Fig 2). The same lag effect was seen for diastolic blood pressure variability (HR for any stroke [lag 3] 1.21, 95% CI 1.13–1.29, *p <* 0.001; HR [lag 6] 1.32, 95% CI 1.20–1.44, *p <* 0.001; HR [lag 9] 1.21, 95% CI 1.04–1.37, *p =* 0.02), except for the association with hemorrhagic stroke, which remained stable over longer time intervals. Using model I in the blood pressure variability analysis (S1 Table), including the lag analysis (S2 Table), yielded similar results. Results from the unadjusted analyses (S3 and S4 Tables) also did not differ.

**Table 2:** Association between systolic blood pressure variability and risk of incident any stroke, ischemic stroke, hemorrhagic stroke, and unspecified stroke.

Outcome	*n/N*	HR per SD		Tertile 1 HR	Tertile 2		Tertile 3	
		HR (95% CI)	*p*-Value		HR (95% CI)	*p*-Value	HR (95% CI)	*p*-Value
***SBP variability***								
Any stroke	971/9,958	**1.11 (1.05–1.18)**	**<0.001**	1 (ref)	1.00 (0.85–1.18)	0.99	1.15 (0.98–1.35)	0.08
Ischemic stroke	641/9,958	1.04 (0.96–1.13)	0.35	1 (ref)	0.96 (0.79–1.17)	0.69	1.02 (0.84–1.24)	0.86
Hemorrhagic stroke	89/9,958	**1.27 (1.05–1.54)**	**0.02**	1 (ref)	0.74 (0.43–1.26)	0.26	1.17 (0.71–1.94)	0.54
Unspecified stroke	241/9,958	**1.21 (1.09–1.34)**	**<0.001**	1 (ref)	1.34 (0.93–1.93)	0.12	**1.64 (1.16–2.31)**	**<0.001**
***DBP variability***								
Any stroke	971/9,955	**1.08 (1.01–1.14)**	**0.02**	1 (ref)	0.86 (0.73–1.02)	0.08	1.03 (0.88–1.21)	0.69
Ischemic stroke	641/9,955	1.02 (0.94–1.10)	0.69	1 (ref)	0.89 (0.73–1.08)	0.23	0.88 (0.72–1.07)	0.19
Hemorrhagic stroke	89/9,955	1.01 (0.79–1.30)	0.92	1 (ref)	0.72 (0.42–1.25)	0.24	1.29 (0.78–2.14)	0.32
Unspecified stroke	241/9,955	**1.19 (1.08–1.31)**	**<0.001**	1 (ref)	0.86 (0.59–1.24)	0.41	**1.43 (1.03–1.99)**	**0.03**

Tertiles 1, 2, and 3 for SBP variability were <1.4%/year, 1.4%–3.4%/year, and >3.4%/year, respectively. Tertiles 1, 2, and 3 for DBP variability were <1.5%/year, 1.5%–3.6%/year, and >3.6%/year, respectively. Values in bold are statistically significant. Adjusted for age, sex, mean SBP or DBP, education, body mass index, alcohol consumption, smoking, cholesterol, high-density lipoprotein cholesterol, lipid-lowering medication use, blood-pressure-lowering medication use, anti-thrombotic medication use, type 2 diabetes mellitus, atrial fibrillation, prior coronary heart disease, and type of blood pressure measuring device. Standard deviation of variance of each tertile for SBP: 0.004 (tertile 1), 0.04 (tertile 2), 0.04 (tertile 3). Standard deviation of variance of each tertile for DBP: 0.004 (tertile 1), 0.006 (tertile 2), 0.05 (tertile 3). DBP, diastolic blood pressure; *n*, number of participants with incident stroke; *N*, total number of participants at risk; SBP, systolic blood pressure; SD, standard deviation.

**Table 3:** Association between blood pressure variability and incident stroke, ischemic stroke, hemorrhagic stroke, and unspecified stroke using different lag periods.

Lag period (years)	Any stroke			Ischemic stroke			Hemorrhagic stroke			Unspecified stroke		
	*n/N*	HR (95% CI)	*p*-Value	*n/N*	HR (95% CI)	*p*-Value	*n/N*	HR (95% CI)	*p*-Value	*n/N*	HR (95% CI)	*p*-Value
***SBP variability***												
3	541/7,241	**1.29 (1.21–1.36)**	**<0.001**	351/7,241	**1.15 (1.04–1.25)**	**0.01**	56/7,241	1.27 (0.99–1.52)	0.06	134/7,241	**1.56 (1.44–1.68)**	**<0.001**
6	212/4,862	**1.47 (1.35–1.59)**	**<0.001**	133/4,862	**1.32 (1.18–1.46)**	**<0.001**	15/4,862	**1.66 (1.26–2.05)**	**0.01**	64/4,862	**1.59 (1.43–1.76)**	**<0.001**
9	118/1,593	**1.38 (1.24–1.51)**	**<0.001**	78/1,593	**1.30 (1.11–1.49)**	**0.01**	11/1,593	**1.49 (1.15–1.82)**	**0.02**	29/1,593	**1.32 (1.07–1.57)**	**0.03**
***DBP variability***												
3	541/7,238	**1.21 (1.13–1.29)**	**<0.001**	351/7,238	1.10 (0.99–1.21)	0.09	56/7,238	1.10 (0.85–1.36)	0.46	134/7,238	**1.48 (1.35–1.62)**	**<0.001**
6	212/4,859	**1.32 (1.20–1.44)**	**<0.001**	133/4,859	**1.20 (1.04–1.36)**	**0.02**	15/4,859	1.14 (0.64–1.64)	0.63	64/4,859	**1.56 (1.35–1.76)**	**<0.001**
9	118/1,591	**1.21 (1.04–1.37)**	**0.02**	78/1,591	1.12 (0.91–1.32)	0.29	11/1,591	0.91 (0.40–1.42)	0.73	29/1,591	**1.55 (1.24–1.85)**	**<0.001**

The estimates represent the HR of incident stroke per standard deviation increase of systolic or diastolic blood pressure variability. Values in bold are statistically significant. Adjusted for age, sex, mean SBP or DBP, and propensity score. CI, confidence interval; DBP, diastolic blood pressure; HR, hazard ratio; *n*, number of participants with incident stroke; *N*, total number of participants at risk; SBP, systolic blood pressure.

### Rise or fall of blood pressure

Both rise and fall of systolic blood pressure were associated with an increased risk of any stroke, but fall of blood pressure showed the strongest associations (Table 4). Regarding stroke subtypes, the strongest association was found for incident unspecified stroke (HR lowest tertile [fall] 1.88, 95% CI 1.31–2.70, *p <* 0.001; HR highest tertile [rise] 1.50, 95% CI 1.04–2.17, *p =* 0.03). An association with incident hemorrhagic stroke was also present but was not significant (HR lowest tertile [fall] 1.39, 95% CI 0.81–2.38, *p =* 0.24; HR highest tertile [rise] 1.45, 95% CI 0.85–2.47, *p =* 0.17). The analyses of rise and fall of diastolic blood pressure showed much weaker associations, with a significant association only between fall of diastolic blood pressure and unspecified stroke (HR lowest tertile [fall] 1.43, 95% CI 1.01–2.01, *p =* 0.04). The lag-specific analyses showed stronger associations between rise and fall of systolic blood pressure and incident stroke, with the strongest associations being with incident any stroke for the 9-year lag period (HR lowest tertile [fall] 2.16, 95% CI 1.58–2.73, *p =* 0.01; HR highest tertile [rise] 1.88, 95% CI 1.32–2.43, *p =* 0.03) (Table 5). Regarding diastolic blood pressure, only fall of diastolic blood pressure was associated with any stroke when longer time intervals were used. Using model I for the analysis of rise and fall of blood pressure (S5 Table), including the lag analysis (S6 Table), yielded similar results. Results from the unadjusted analyses (S7 and S8 Tables) also did not differ.

**Table 4:** Association between rise and fall of blood pressure and incident stroke, ischemic stroke, hemorrhagic stroke, and unspecified stroke.

Outcome	*n/N*	Tertile 1		Tertile 2 HR	Tertile 3	
		HR (95% CI)	*p*-Value		HR (95% CI)	*p*-Value
***Systolic blood pressure***						
Any stroke	971/9,958	**1.27 (1.08–1.49)**	**<0.001**	1 (ref)	1.12 (0.95–1.32)	0.17
Ischemic stroke	641/9,958	1.09 (0.90–1.33)	0.38	1 (ref)	0.99 (0.82–1.21)	0.95
Hemorrhagic stroke	89/9,958	1.39 (0.81–2.38)	0.24	1 (ref)	1.45 (0.85–2.47)	0.17
Unspecified stroke	241/9,958	**1.88 (1.31–2.70)**	**<0.001**	1 (ref)	**1.50 (1.04–2.17)**	**0.03**
***Diastolic blood pressure***						
Any stroke	971/9,955	1.10 (0.94–1.28)	0.26	1 (ref)	0.88 (0.74–1.03)	0.11
Ischemic stroke	641/9,955	1.04 (0.86–1.26)	0.67	1 (ref)	0.82 (0.67–1.00)	0.06
Hemorrhagic stroke	89/9,955	0.79 (0.47–1.33)	0.38	1 (ref)	0.84 (0.51–1.40)	0.50
Unspecified stroke	241/9,955	**1.43 (1.01–2.01)**	**0.04**	1 (ref)	1.10 (0.77–1.58)	0.59

Tertiles 1, 2, and 3 for systolic blood pressure were <−0.4%/year, −0.4% to 2.1%/year, and >2.1%/year, respectively. Tertiles 1, 2, and 3 for diastolic blood pressure were <−0.8%/year, −0.8% to 2.0%/year, and >2.0%/year, respectively. Values in bold are statistically significant. Adjusted for age, sex, mean systolic or diastolic blood pressure, education, body mass index, alcohol consumption, smoking, cholesterol, high-density lipoprotein cholesterol, lipid-lowering medication use, blood-pressure-lowering medication use, anti-thrombotic medication use, type 2 diabetes mellitus, atrial fibrillation, prior coronary heart disease, and type of blood pressure measuring device. Standard deviation of variance of each tertile for systolic blood pressure: 0.04 (tertile 1), 0.03 (tertile 2), 0.03 (tertile 3). Standard deviation of variance of each tertile for diastolic blood pressure: 0.04 (tertile 1), 0.008 (tertile 2), 0.04 (tertile 3). *n*, number of participants with incident stroke; *N*, total study population; ref, reference.

**Table 5:** Association between rise and fall of blood pressure and incident any stroke using different lag periods.

Lag period (years)	*n/N*	Tertile 1		Tertile 2 HR	Tertile 3	
		HR (95% CI)	*p*-Value		HR (95% CI)	*p*-Value
***Systolic blood pressure***						
3	541/7,241	**1.54 (1.32–1.75)**	**<0.001**	1 (ref)	**1.31 (1.09–1.52)**	**0.01**
6	212/4,862	**2.07 (1.68–2.45)**	**<0.001**	1 (ref)	**1.80 (1.43–2.18)**	**<0.001**
9	118/1,593	**2.16 (1.58–2.73)**	**0.01**	1 (ref)	**1.88 (1.32–2.43)**	**0.03**
***Diastolic blood pressure***						
3	541/7,238	**1.46 (1.25–1.67)**	**<0.001**	1 (ref)	1.00 (0.78–1.22)	0.99
6	212/4,859	**1.49 (1.14–1.84)**	**0.03**	1 (ref)	1.19 (0.84–1.54)	0.34
9	118/1,591	1.38 (0.89–1.87)	0.19	1 (ref)	1.12 (0.62–1.62)	0.67

Values in bold are statistically significant. Adjusted for age, sex, mean systolic or diastolic blood pressure, and propensity score. CI, confidence interval; HR, hazard ratio; *n*, number of participants with incident any stroke; *N*, total number of participants at risk; ref, reference.

### Sensitivity analyses

**Fig 3:**
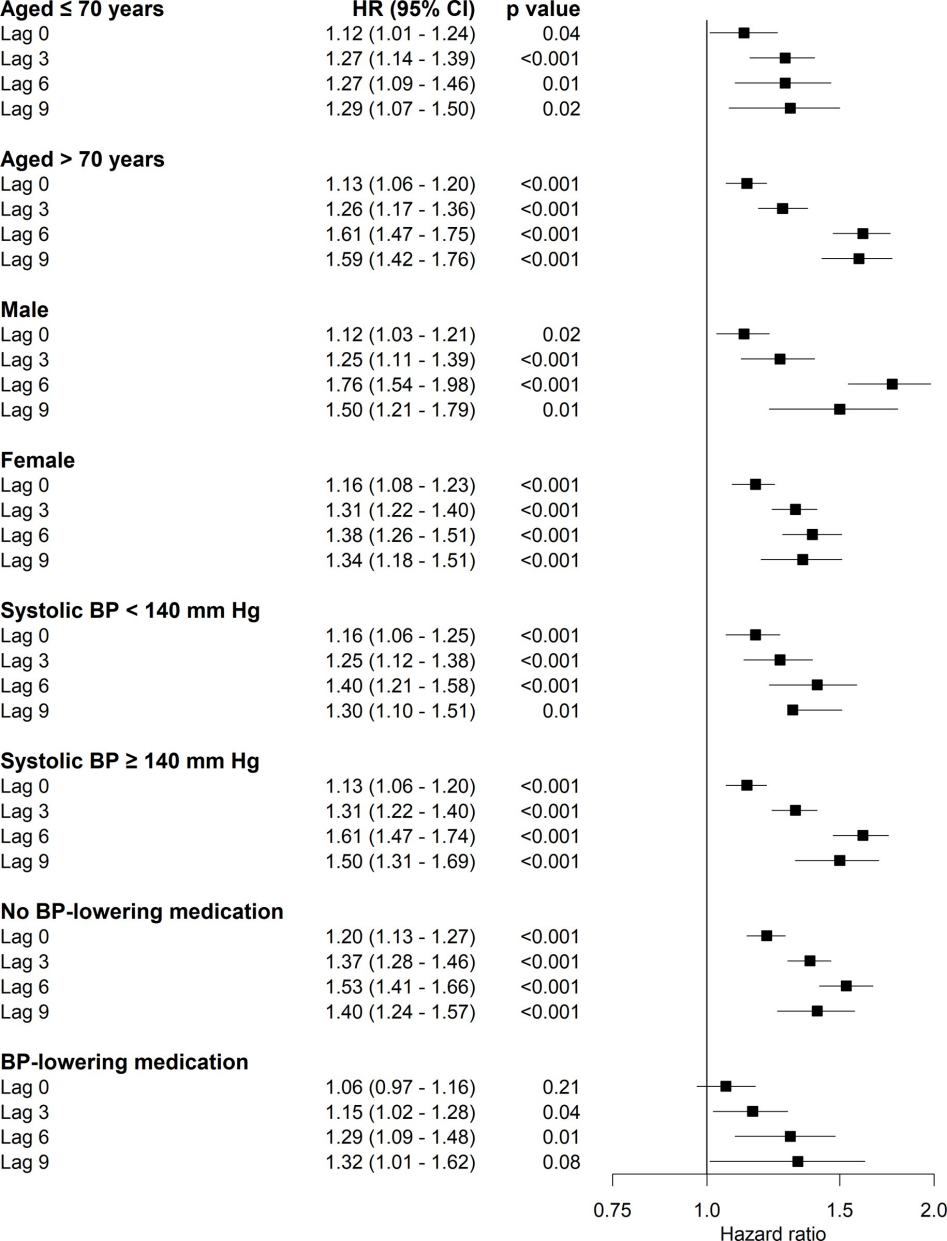
Stratified analyses of the association between systolic blood pressure (BP) variability and incident any stroke using different lag periods. The black squares represent the hazard ratio (HR) of incident any stroke per SD increase in systolic blood pressure variability. The error bars are the 95% confidence intervals (CIs) around the hazard ratios. The models were adjusted for age, sex, mean systolic blood pressure, and propensity score.

We found no significant interaction between systolic blood pressure variability and baseline systolic blood pressure for all stroke outcomes (*p*-value for interaction for any, ischemic, hemorrhagic, and unspecified stroke, respectively: 0.67, 0.45, 0.78, and 0.50). The association between systolic blood pressure variability and risk of any stroke was especially pronounced in participants aged >70 years (HR [lag 0] 1.13, 95% CI 1.06–1.20, *p <* 0.001; HR [lag 3] 1.26, 95% CI 1.17–1.36, *p <* 0.001; HR [lag 6] 1.61, 95% CI 1.47–1.75, *p <* 0.001; HR [lag 9] 1.59, 95% CI 1.42–1.76, *p <* 0.001) (Fig 3). Furthermore, the association was also stronger in participants who did not use blood-pressure-lowering medication (HR [lag 0] 1.20, 95% CI 1.13–1.27, *p <* 0.001; HR [lag 3] 1.37, 95% CI 1.28–1.46, *p <* 0.001; HR [lag 6] 1.53, 95% CI 1.41–1.66, *p <* 0.001; HR [lag 9] 1.40, 95% CI 1.24–1.57, *p <* 0.001). We found no differences between men and women or between individuals with different baseline systolic blood pressure levels. When repeating the analysis using complete cases only (7,550 participants), 59% of the strokes remained in the analysis. In this analysis, we did not find an association between systolic blood pressure variation and stroke (S9 Table).

## Discussion

We found that a larger visit-to-visit variation in blood pressure was associated with an increased risk of stroke, in particular for unspecified and hemorrhagic strokes. These associations were more pronounced for variation in systolic than diastolic blood pressure and were stronger as the interval between blood pressure variability measurement and diagnosis of stroke increased. Additional sensitivity analyses with lag time periods supported these associations. Finally, a higher risk of stroke was associated with both rises and falls in systolic blood pressure, suggesting that larger variation, rather than the direction, was associated with stroke risk.

Previous population-based studies on visit-to-visit blood pressure variability and stroke risk within selected participants have yielded contradictory results. A study of 470 chronic kidney disease patients in the CMERC-HI cohort found that systolic blood pressure variability was strongly associated with a composite outcome of cardiovascular events including stroke and all-cause mortality [11]. Diastolic blood pressure variability was non-significantly associated with these outcomes. The Three-City Cohort Study, including 5,951 hypertensive patients, did not find an association between systolic and diastolic blood pressure variability and stroke risk, although no risk estimates were provided [13]. Finally, in the Cardiovascular Health Study, systolic and diastolic blood pressure were associated with mortality and myocardial infarction but not with stroke in individuals with either stable (*n =* 1,095) or no (*n =* 1,642) use of antihypertensive medication [12]. Possible explanations for this finding may have been the small number of stroke events, limiting study power, and the selection of healthier individuals who survived event-free over the course of 5 years without any changes in their antihypertensive medication use. Cohort studies within trials of hypertensive patients, although less generalizable due to the trial selection criteria and inclusion of high-risk individuals, did find higher stroke risks in patients with increased blood pressure variability, with overall stronger associations for systolic than diastolic blood pressure variability [6–10].

We found that systolic blood pressure variability was associated with increased risk of hemorrhagic and unspecified stroke, whereas diastolic blood pressure variability was not associated with hemorrhagic stroke. Of the abovementioned studies that found an association between blood pressure variability and stroke, 3 studies stratified by stroke subtype [7,9,10]. One study found the same risk for ischemic and hemorrhagic stroke in those with larger systolic blood pressure variability [10], whereas the other 2 studies found that systolic and diastolic blood pressure variability were associated with the risk of ischemic stroke specifically, and were not, or were to a lesser extent, associated with the risk of hemorrhagic or unspecified stroke [7,9]. Strokes in our study population were classified as unspecified when neuroimaging was missing. Patients with unspecified stroke represent a frail patient group, with individuals who are older, more often live in nursing homes, and frequently have additional comorbidities such as dementia and diabetes mellitus [22]. The frailty of this high-risk population may not have been captured fully by adjusting for cardiovascular risk factors, which could explain our strong risk estimates for unspecified stroke.

Several pathophysiological mechanisms have been proposed to underlie the association between blood pressure variability and stroke. Repeated fluctuations in blood flow, amplified by blood pressure variability, increase mechanical stress on the arterial walls, prompting structural vascular remodeling such as arterial stiffening [3,23,24], endothelial dysfunction [2,25], platelet activation [26,27], and inflammation [28,29]. Cerebrovascular microvasculature is especially vulnerable to these fluctuations due to its lower vascular resistance and upstream vasodilation, possibly leading to small vessel disease [2,24,25,30]. Indeed, blood pressure variability has been associated with cerebral small vessel disease and in particular white matter hyperintensities [16,30,31]. Our study further emphasizes the importance of blood pressure variability, rather than the direction of blood pressure change, since both rise and fall of systolic blood pressure were associated with stroke risk, and the increased risk of systolic blood pressure variability was still present in participants with systolic blood pressure of 140 mm Hg or higher. Increasing age and arterial stiffness further limit the ability of baroreceptors to buffer these detrimental fluctuations in blood pressure [2,3]. This may explain why, in our sensitivity analyses, we found that higher systolic blood pressure variability was associated with stroke risk especially in older participants.

Another explanation that has been proposed for the association of blood pressure variability and stroke is that accumulating cerebrovascular pathology and ischemia could be a common cause of both stroke and blood pressure variability, via impaired autonomic control [32]. We addressed this not only by adjusting our models for major cardiovascular risk factors, but also by increasing the time interval between measurement of blood pressure variability and assessment of stroke. The higher stroke risk with increasing time intervals suggests that blood pressure variability is indeed a long-term and independent risk factor of stroke.

Several limitations of this study need to be taken into consideration. We assumed a constant rate of blood pressure variability between visits over a period of several years, possibly introducing random measurement error, which could attenuate our findings. Due to an average interval of 4 years between visits, we may have not captured blood pressure variability as timely as in other studies with more frequent visits. Due to this interval between visits, we based blood pressure variability on 2 measures to prevent immortal time bias and healthy volunteer effects. Moreover, although use of blood-pressure-lowering medication was updated during each visit to the research center, we could not account for initiation of antihypertensive medication in between visits. Despite these limitations, we found consistent and strong dose–response associations over several time intervals. Similarly, a small number of our participants (*n =* 145) missed an examination round. Blood pressure variability could therefore not be updated for a longer period for these participants, introducing heterogeneity in our measurements. However, this group was a small percentage (1.5%) of our study population, and excluding this group may introduce selection bias of healthier participants. Furthermore, although we adjusted for mean systolic blood pressure and blood-pressure-lowering medication use, we cannot rule out the possibility of some individuals with severe hypertension being successfully treated and as a result being misclassified as having a fall of blood pressure. Finally, we used a multiple imputation approach to account for missing data on the covariates since we could not satisfy the strict condition of missing completely at random (MCAR) for an adjusted complete case analysis [22,33]. However, multiple imputation analysis also has its limitations since it requires a missing at random (MAR) assumption. Although we believe this assumption is justifiable for our data, it is difficult to show the differences between data that are MAR versus missing not at random (MNAR). Nevertheless, our unadjusted analyses that did not include imputed data showed similar results. Complete case analysis showed no significant associations with stroke. Although we included this analysis (S9 Table), we fully realize that this analysis has an even stricter requirement, that is, MCAR. In any population-based study, including the Rotterdam Study, missingness is never thought to be “completely at random,” so we feel that the MCAR assumption that is required for a complete case analysis is not justifiable. In addition, when including only complete cases, 76% of the original study population remained, and more importantly, 59% of those with any stroke remained in the analysis. Moreover, this percentage was different among the various subtypes, with a larger percentage of unspecified stroke being lost in comparison to the other subtypes; the following percentages remained in the analysis: 63% of ischemic, 64% of hemorrhagic, and 45% of unspecified strokes. This already provides some hints that the MCAR assumption for a complete case scenario is not justifiable and multiple imputation is more appropriate.

Strengths of this study include its population-based setting with long follow-up, continuous assessment of stroke incidence, the time-varying inclusion of blood pressure variability and confounders in our models, and the use of increasing time intervals from 3 to 9 years between variability measurement and stroke assessment to reduce reverse causation.

Future studies should further unravel underlying pathways and determine whether reducing blood pressure variability results in a lower stroke risk.

### Conclusion

In this study, we found that larger visit-to-visit blood pressure variation was associated with an increased risk of stroke and its hemorrhagic and unspecified subtypes, independent of the direction of variation or level of blood pressure. Moreover, the association with the risk of stroke and its subtypes was stronger as the interval between measurement of blood pressure variation and diagnosis of stroke increased. Our findings suggest that blood pressure variability is an independent risk factor for stroke in the general population.

## Supporting information

S1 Table Association between systolic blood pressure variability and risk of incident any stroke, ischemic stroke, hemorrhagic stroke, and unspecified stroke—adjusted for age, sex, and mean systolic or diastolic blood pressure. (DOCX)

S2 Table Association between blood pressure variability and incident stroke, ischemic stroke, hemorrhagic stroke, and unspecified stroke using different lag periods—adjusted for age, sex, and mean systolic or diastolic blood pressure. (DOCX)

S3 Table Association between systolic blood pressure variability and risk of incident any stroke, ischemic stroke, hemorrhagic stroke, and unspecified stroke—unadjusted. (DOCX)

S4 Table Association between blood pressure variability and incident stroke, ischemic stroke, hemorrhagic stroke, and unspecified stroke using different lag periods—unadjusted. (DOCX)

S5 Table Association between rise and fall of blood pressure and incident stroke, ischemic stroke, hemorrhagic stroke, and unspecified stroke—adjusted for age, sex, and mean systolic or diastolic blood pressure. (DOCX)

S6 Table Association between rise and fall of blood pressure and incident any stroke using different lag periods—adjusted for age, sex, and mean systolic or diastolic blood pressure. (DOCX)

S7 Table Association between rise and fall of blood pressure and incident stroke, ischemic stroke, hemorrhagic stroke, and unspecified stroke—unadjusted. (DOCX)

S8 Table Association between rise and fall of blood pressure and incident any stroke using different lag periods—unadjusted. (DOCX)

S9 Table Association between systolic blood pressure variability and risk of incident any stroke, ischemic stroke, hemorrhagic stroke, and unspecified stroke using complete cases only. (DOCX)

S1 Text STROBE checklist. (DOCX)

## Abbreviations

DBP: diastolic blood pressure
HDL: cholesterol, high-density lipoprotein cholesterol
HR: hazard ratio
MAR: missing at random
MCAR: missing completely at random
SBP: systolic blood pressure
