# Supplementary material for: Visit-to-visit blood pressure variability and the risk of stroke in the Netherlands: A population-based cohort study
Source: PLoS Med. 2022 Mar 17;19(3):e1003942. doi: 10.1371/journal.pmed.1003942 (PMC8929650; doi:10.1371/journal.pmed.1003942)
Supplement: S1 Table — (DOCX) [file pmed.1003942.s001.docx]

**Table S1.** Association between systolic blood pressure variability and risk of incident any stroke, ischemic stroke, haemorrhagic stroke, and unspecified stroke (adjusted for age, sex and mean systolic or diastolic blood pressure).

|  |  | n/N |  |  |  | | | | Hazard ratio (95% confidence interval) | | | |  |
| --- | --- | --- | --- | --- | --- | --- | --- | --- | --- | --- | --- | --- | --- |
|  |  |  |  | per SD | | p value |  | Tertile 1  (<1.4%/year) | | Tertile 2  (1.4-3.4%/year) | p value | Tertile 3  (>3.4%/year) | p value |
| *SBP variability* |  |  |  |  | |  |  |  | |  |  |  |  |
| Any stroke |  | 971/9958 |  | **1.15 (1.08 – 1.21)** | | <0.001 |  | 1 [ref] | | 1.03 (0.88 – 1.21) | 0.71 | **1.27 (1.09 – 1.49)** | **<0.001** |
| Ischemic stroke |  | 641/9958 |  | 1.07 (0.99 – 1.16) | | 0.08 |  | 1 [ref] | | 0.98 (0.81 – 1.20) | 0.87 | 1.12 (0.92 – 1.35) | 0.27 |
| Haemorrhagic stroke |  | 89/9958 |  | **1.22 (1.01 – 1.48)** | | **0.03** |  | 1 [ref] | | 0.75 (0.44 – 1.28) | 0.29 | 1.12 (0.68 – 1.84) | 0.66 |
| Unspecified stroke |  | 241/9958 |  | **1.27 (1.15 – 1.40)** | | **<0.001** |  | 1 [ref] | | 1.41 (0.98 – 2.03) | 0.07 | **1.95 (1.38 – 2.74)** | **<0.001** |
|  |  |  |  |  | |  |  |  | |  |  |  |  |
| *DBP variability* |  |  |  |  | |  |  | (<1.5%/year) | | (1.5-3.6%/year) |  | (>3.6%/year) |  |
| Any stroke |  | 971/9955 |  | **1.11 (1.05 – 1.18)** | | **<0.001** |  | 1 [ref] | | 0.89 (0.75 – 1.04) | 0.15 | 1.13 (0.97 – 1.32) | 0.12 |
| Ischemic stroke |  | 641/9955 |  | 1.05 (0.97 – 1.13) | | 0.26 |  | 1 [ref] | | 0.91 (0.75 – 1.10) | 0.32 | 0.96 (0.79 – 1.16) | 0.67 |
| Haemorrhagic stroke |  | 89/9955 |  | 0.98 (0.77 – 1.26) | | 0.90 |  | 1 [ref] | | 0.70 (0.41 – 1.19) | 0.19 | 1.22 (0.74 – 1.99) | 0.43 |
| Unspecified stroke |  | 241/9955 |  | **1.25 (1.14 – 1.37)** | | **<0.001** |  | 1 [ref] | | 0.93 (0.64 – 1.35) | 0.70 | **1.68 (1.21 – 2.34)** | **<0.001** |

Adjusted for age, sex and mean systolic or diastolic blood pressure. Standard deviation of variance of each tertile for systolic blood pressure: 0.004 (tertile 1), 0.04 (tertile 2), 0.04 (tertile 3). Standard deviation of variance of each tertile for diastolic blood pressure: 0.004 (tertile 1), 0.006 (tertile 2), 0.05 (tertile 3). Abbreviations: DBP; diastolic blood pressure, n; number of participants with incident stroke, N; total number of participants at risk, SBP; systolic blood pressure, SD; standard deviation.
