# Supplementary material for: Visit-to-visit blood pressure variability and the risk of stroke in the Netherlands: A population-based cohort study
Source: PLoS Med. 2022 Mar 17;19(3):e1003942. doi: 10.1371/journal.pmed.1003942 (PMC8929650; doi:10.1371/journal.pmed.1003942)
Supplement: S2 Table — (DOCX) [file pmed.1003942.s002.docx]

**Table S2.** Association between blood pressure variability and incident stroke, ischemic stroke, haemorrhagic stroke and unspecified stroke using different lag periods (adjusted for age, sex and mean systolic or diastolic blood pressure).

| Lag period (years) |  | Any stroke | | | |  |  | Ischemic stroke | | | |  |  | Hemorrhagic stroke | | |  |  | Unspecified stroke | | |  |  |
| --- | --- | --- | --- | --- | --- | --- | --- | --- | --- | --- | --- | --- | --- | --- | --- | --- | --- | --- | --- | --- | --- | --- | --- |
|  |  | n/N | HR (95% CI) | | | p value |  | n/N | HR (95% CI) | | | p value |  | n/N | HR (95% CI) | | p value |  | n/N | HR (95% CI) | | p value |  |
| *SBP variability* | | | |  |  |  |  | | |  |  |  |  |  | |  |  |  |  | |  | | |
| 3 |  | 541/7241 | **1.31 (1.24 – 1.39)** | | | **<0.001** |  | 351/7241 | **1.16 (1.06 – 1.27)** | | | **<0.001** |  | 56/7241 | 1.28 (1.03 – 1.54) | | 0.05 |  | 134/7241 | **1.57 (1.46 – 1.68)** | | **<0.001** |  |
| 6 |  | 212/4862 | **1.47 (1.36 – 1.59)** | | | **<0.001** |  | 133/4862 | **1.32 (1.18 – 1.46)** | | | **<0.001** |  | 15/4862 | **1.71 (1.37 – 2.05)** | | **<0.001** |  | 64/4862 | **1.59 (1.45 – 1.72)** | | **<0.001** |  |
| 9 |  | 118/1593 | **1.38 (1.24 – 1.51)** | | | **<0.001** |  | 78/1593 | **1.33 (1.16 – 1.50)** | | | **<0.001** |  | 11/1593 | **1.67 (1.32 – 2.01)** | | **<0.001** |  | 29/1593 | **1.32 (1.06 – 1.57)** | | **0.03** |  |
|  |  |  |  | | |  |  |  |  | | |  |  |  |  | |  |  |  |  | |  |  |
| *DBP variability* | | | |  |  |  |  | | |  |  |  |  |  | |  |  |  |  | |  | | |
| 3 |  | 541/7238 | **1.24 (1.16 – 1.31)** | | | **<0.001** |  | 351/7238 | **1.14 (1.03 – 1.24)** | | | **0.02** |  | 56/7238 | 1.08 (0.86 – 1.31) | | 0.50 |  | 134/7238 | **1.45 (1.35 – 1.56)** | | **<0.001** |  |
| 6 |  | 212/4859 | **1.31 (1.21 – 1.42)** | | | **<0.001** |  | 133/4859 | **1.21 (1.07 – 1.36)** | | | **0.01** |  | 15/4859 | 1.14 (0.68 – 1.61) | | 0.63 |  | 64/4859 | **1.47 (1.31 – 1.63)** | | **<0.001** |  |
| 9 |  | 118/1591 | **1.20 (1.05 – 1.35)** | | | **0.02** |  | 78/1591 | 1.12 (0.92 – 1.31) | | | 0.28 |  | 11/1591 | 0.94 (0.46 – 1.42) | | 0.81 |  | 29/1591 | **1.49 (1.26 – 1.71)** | | **<0.001** |  |

The estimates represent the hazard ratio of incident stroke per standard deviation increase of systolic blood pressure variability. Adjusted for age, sex and mean systolic or diastolic blood pressure. Abbreviations: DBP; diastolic blood pressure, CI; confidence interval, HR; hazard ratio, n; number of participants with incident stroke, N; total number of participants at risk, SBP; systolic blood pressure.
