# Supplementary material for: Visit-to-visit blood pressure variability and the risk of stroke in the Netherlands: A population-based cohort study
Source: PLoS Med. 2022 Mar 17;19(3):e1003942. doi: 10.1371/journal.pmed.1003942 (PMC8929650; doi:10.1371/journal.pmed.1003942)
Supplement: S3 Table — (DOCX) [file pmed.1003942.s003.docx]

**Table S3.** Association between systolic blood pressure variability and risk of incident any stroke, ischemic stroke, haemorrhagic stroke, and unspecified stroke (unadjusted).

|  |  | n/N |  |  |  | | | | Hazard ratio (95% confidence interval) | | | |  |
| --- | --- | --- | --- | --- | --- | --- | --- | --- | --- | --- | --- | --- | --- |
|  |  |  |  | per SD | | p value |  | Tertile 1  (<1.4%/year) | | Tertile 2  (1.4-3.4%/year) | p value | Tertile 3  (>3.4%/year) | p value |
| *SBP variability* |  |  |  |  | |  |  |  | |  |  |  |  |
| Any stroke |  | 971/9958 |  | **1.14 (1.08 – 1.21)** | | <0.001 |  | 1 [ref] | | 1.04 (0.89 – 1.23) | 0.60 | **1.33 (1.14 – 1.56)** | **<0.001** |
| Ischemic stroke |  | 641/9958 |  | 1.07 (0.99 – 1.15) | | 0.10 |  | 1 [ref] | | 0.99 (0.82 – 1.21) | 0.93 | 1.14 (0.94 – 1.38) | 0.17 |
| Haemorrhagic stroke |  | 89/9958 |  | **1.21 (1.01 – 1.45)** | | **0.04** |  | 1 [ref] | | 0.75 (0.44 – 1.28) | 0.29 | 1.13 (0.69 – 1.85) | 0.62 |
| Unspecified stroke |  | 241/9958 |  | **1.28 (1.17 – 1.40)** | | **<0.001** |  | 1 [ref] | | **1.46 (1.01 – 2.09)** | **0.04** | **2.23 (1.59 – 3.13)** | **<0.001** |
|  |  |  |  |  | |  |  |  | |  |  |  |  |
| *DBP variability* |  |  |  |  | |  |  | (<1.5%/year) | | (1.5-3.6%/year) |  | (>3.6%/year) |  |
| Any stroke |  | 971/9955 |  | **1.12 (1.06 – 1.19)** | | **<0.001** |  | 1 [ref] | | 0.91 (0.78 – 1.07) | 0.28 | **1.23 (1.05 – 1.43)** | 0.01 |
| Ischemic stroke |  | 641/9955 |  | 1.05 (0.97 – 1.13) | | 0.24 |  | 1 [ref] | | 0.92 (0.76 – 1.11) | 0.38 | 0.99 (0.82 – 1.20) | 0.93 |
| Haemorrhagic stroke |  | 89/9955 |  | 0.97 (0.76 – 1.24) | | 0.80 |  | 1 [ref] | | 0.70 (0.41 – 1.19) | 0.19 | 1.19 (0.73 – 1.95) | 0.48 |
| Unspecified stroke |  | 241/9955 |  | **1.30 (1.19 – 1.41)** | | **<0.001** |  | 1 [ref] | | 1.03 (0.71 – 1.48) | 0.88 | **2.15 (1.55 – 2.96)** | **<0.001** |

Standard deviation of variance of each tertile for systolic blood pressure: 0.004 (tertile 1), 0.04 (tertile 2), 0.04 (tertile 3). Standard deviation of variance of each tertile for diastolic blood pressure: 0.004 (tertile 1), 0.006 (tertile 2), 0.05 (tertile 3). Abbreviations: DBP; diastolic blood pressure, n; number of participants with incident stroke, N; total number of participants at risk, SBP; systolic blood pressure, SD; standard deviation.
