# Supplementary material for: Visit-to-visit blood pressure variability and the risk of stroke in the Netherlands: A population-based cohort study
Source: PLoS Med. 2022 Mar 17;19(3):e1003942. doi: 10.1371/journal.pmed.1003942 (PMC8929650; doi:10.1371/journal.pmed.1003942)
Supplement: S4 Table — (DOCX) [file pmed.1003942.s004.docx]

**Table S4.** Association between blood pressure variability and incident stroke, ischemic stroke, haemorrhagic stroke and unspecified stroke using different lag periods (unadjusted).

| Lag period (years) |  | Any stroke | | | |  |  | Ischemic stroke | | | |  |  | Hemorrhagic stroke | | |  |  | Unspecified stroke | | |  |  |
| --- | --- | --- | --- | --- | --- | --- | --- | --- | --- | --- | --- | --- | --- | --- | --- | --- | --- | --- | --- | --- | --- | --- | --- |
|  |  | n/N | HR (95% CI) | | | p value |  | n/N | HR (95% CI) | | | p value |  | n/N | HR (95% CI) | | p value |  | n/N | HR (95% CI) | | p value |  |
| *SBP variability* | | | |  |  |  |  | | |  |  |  |  |  | |  |  |  |  | |  | | |
| 3 |  | 541/7241 | **1.28 (1.21 – 1.35)** | | | **<0.001** |  | 351/7241 | **1.14 (1.04 – 1.24)** | | | **0.01** |  | 56/7241 | 1.24 (0.99 – 1.49) | | 0.09 |  | 134/7241 | **1.53 (1.44 – 1.62)** | | **<0.001** |  |
| 6 |  | 212/4862 | **1.46 (1.35 – 1.57)** | | | **<0.001** |  | 133/4862 | **1.39 (1.26 – 1.52)** | | | **<0.001** |  | 15/4862 | **1.61 (1.35 – 1.86)** | | **<0.01** |  | 64/4862 | **1.52 (1.37 – 1.67)** | | **<0.001** |  |
| 9 |  | 118/1593 | **1.39 (1.26 – 1.51)** | | | **<0.001** |  | 78/1593 | **1.38 (1.23 – 1.54)** | | | **<0.001** |  | 11/1593 | **1.61 (1.37 – 1.85)** | | **0.001** |  | 29/1593 | 1.25 (0.99 – 1.50) | | 0.07 |  |
|  |  |  |  | | |  |  |  |  | | |  |  |  |  | |  |  |  |  | |  |  |
| *DBP variability* | | | |  |  |  |  | | |  |  |  |  |  | |  |  |  |  | |  | | |
| 3 |  | 541/7238 | **1.22 (1.15 – 1.29)** | | | **<0.001** |  | 351/7238 | **1.12 (1.02 – 1.22)** | | | **0.03** |  | 56/7238 | 1.05 (0.83 – 1.28) | | 0.66 |  | 134/7238 | **1.44 (1.35 – 1.53)** | | **<0.001** |  |
| 6 |  | 212/4859 | **1.30 (1.20 – 1.40)** | | | **<0.001** |  | 133/4859 | **1.25 (1.11 – 1.39)** | | | **<0.01** |  | 15/4859 | 1.14 (0.77 – 1.51) | | 0.50 |  | 64/4859 | **1.40 (1.26 – 1.54)** | | **<0.001** |  |
| 9 |  | 118/1591 | **1.22 (1.09 – 1.36)** | | | **<0.01** |  | 78/1591 | 1.18 (1.00 – 1.37) | | | 0.08 |  | 11/1591 | 0.94 (0.46 – 1.42) | | 0.99 |  | 29/1591 | **1.41 (1.20 – 1.61)** | | **<0.01** |  |

The estimates represent the hazard ratio of incident stroke per standard deviation increase of systolic blood pressure variability. Abbreviations: DBP; diastolic blood pressure, CI; confidence interval, HR; hazard ratio, n; number of participants with incident stroke, N; total number of participants at risk, SBP; systolic blood pressure.
