# Supplementary material for: Visit-to-visit blood pressure variability and the risk of stroke in the Netherlands: A population-based cohort study
Source: PLoS Med. 2022 Mar 17;19(3):e1003942. doi: 10.1371/journal.pmed.1003942 (PMC8929650; doi:10.1371/journal.pmed.1003942)
Supplement: S5 Table — (DOCX) [file pmed.1003942.s005.docx]

**Table S5.** Association between rise and fall of blood pressure and incident stroke, ischemic stroke, haemorrhagic stroke, and unspecified stroke (adjusted for age, sex and mean systolic or diastolic blood pressure).

|  |  | n/N |  |  | Hazard ratio (95% confidence interval) | | | |  |
| --- | --- | --- | --- | --- | --- | --- | --- | --- | --- |
|  |  |  |  | Tertile 1  (<-0.4%/year) | | p value | Tertile 2  (-0.4-2.1%/year) | Tertile 3  (>2.1%) | p value |
| *Systolic blood pressure* |  |  |  |  | |  |  |  |  |
| Any stroke |  | 971/9958 |  | **1.35 (1.15 – 1.59)** | | **<0.001** | 1 [ref] | **1.20 (1.02 – 1.41)** | **0.03** |
| Ischemic stroke |  | 641/9958 |  | 1.16 (0.96 – 1.41) | | 0.13 | 1 [ref] | 1.06 (0.87 – 1.28) | 0.58 |
| Haemorrhagic stroke |  | 89/9958 |  | 1.40 (0.82 – 2.40) | | 0.21 | 1 [ref] | 1.37 (0.81 – 2.32) | 0.24 |
| Unspecified stroke |  | 241/9958 |  | **2.08 (1.45 – 2.98)** | | **<0.001** | 1 [ref] | **1.74 (1.21 – 2.50)** | **<0.001** |
|  |  |  |  |  | |  |  |  |  |
| *Diastolic blood pressure* | | |  | <-0.8%/year) | |  | (-0.08-2.0%/year) | (>2.0%/year) |  |
| Any stroke |  | 971/9955 |  | **1.20 (1.02 – 1.40)** | | **0.02** | 1 [ref] | 0.93 (0.79 – 1.09) | 0.35 |
| Ischemic stroke |  | 641/9955 |  | 1.14 (0.94 – 1.37) | | 0.19 | 1 [ref] | 0.86 (0.71 – 1.05) | 0.14 |
| Haemorrhagic stroke |  | 89/9955 |  | 0.83 (0.50 – 1.38) | | 0.47 | 1 [ref] | 0.80 (0.49 – 1.33) | 0.40 |
| Unspecified stroke |  | 241/9955 |  | **1.62 (1.15 – 2.28)** | | **0.01** | 1 [ref] | 1.25 (0.88 – 1.78) | 0.21 |

Adjusted for age, sex and mean systolic or diastolic blood pressure. Standard deviation of variance of each tertile for systolic blood pressure: 0.04 (tertile 1), 0.03 (tertile 2), 0.03 (tertile 3). Standard deviation of variance of each tertile for diastolic blood pressure: 0.04 (tertile 1), 0.008 (tertile 2), 0.04 (tertile 3). Abbreviations: n; number of participants with incident stroke, N; total study population, ref; reference.
