# Supplementary material for: Visit-to-visit blood pressure variability and the risk of stroke in the Netherlands: A population-based cohort study
Source: PLoS Med. 2022 Mar 17;19(3):e1003942. doi: 10.1371/journal.pmed.1003942 (PMC8929650; doi:10.1371/journal.pmed.1003942)
Supplement: S6 Table — (DOCX) [file pmed.1003942.s006.docx]

**Table S6**. Association between rise and fall of blood pressure and incident any stroke using different lag periods (adjusted for age, sex and mean systolic or diastolic blood pressure).

| Lag period (years) |  |  | Any stroke | | | | |
| --- | --- | --- | --- | --- | --- | --- | --- |
|  |  | n/N | Tertile 1  HR (95% CI) | p value | Tertile 2  HR (95% CI) | Tertile 3  HR (95% CI) | p value |
| *Systolic blood pressure* |  |  |  |  |  |  |  |
| 3 |  | 541/7241 | **1.54 (1.33 – 1.76)** | **<0.001** | 1 [ref] | **1.31 (1.09 – 1.52)** | **0.01** |
| 6 |  | 212/4862 | **2.07 (1.69 – 2.46)** | **<0.001** | 1 [ref] | **1.80 (1.43 – 2.17)** | **<0.001** |
| 9 |  | 118/1593 | **2.19 (1.62 – 2.77)** | **0.01** | 1 [ref] | **1.88 (1.32 – 2.43)** | **0.03** |
|  |  |  |  |  |  |  |  |
| *Diastolic blood pressure* |  |  |  |  |  |  |  |
| 3 |  | 541/7238 | **1.46 (1.25 – 1.67)** | **<0.001** | 1 [ref] | 1.00 (0.78 – 1.22) | 0.99 |
| 6 |  | 212/4859 | **1.53 (1.17 – 1.88)** | **0.02** | 1 [ref] | 1.18 (0.83 – 1.54) | 0.34 |
| 9 |  | 118/1591 | 1.42 (0.93 – 1.91) | 0.16 | 1 [ref] | 1.11 (0.61 – 1.61) | 0.70 |

Adjusted for age, sex and mean systolic or diastolic blood pressure. Abbreviations: CI; confidence interval, HR; hazard ratio, n; number of participants with incident any stroke, N; total number of participants at risk, ref; reference.
