# Supplementary material for: Visit-to-visit blood pressure variability and the risk of stroke in the Netherlands: A population-based cohort study
Source: PLoS Med. 2022 Mar 17;19(3):e1003942. doi: 10.1371/journal.pmed.1003942 (PMC8929650; doi:10.1371/journal.pmed.1003942)
Supplement: S7 Table — (DOCX) [file pmed.1003942.s007.docx]

**Table S7.** Association between rise and fall of blood pressure and incident stroke, ischemic stroke, haemorrhagic stroke, and unspecified stroke (unadjusted).

|  |  | n/N |  |  | Hazard ratio (95% confidence interval) | | | |  |
| --- | --- | --- | --- | --- | --- | --- | --- | --- | --- |
|  |  |  |  | Tertile 1  (<-0.4%/year) | | p value | Tertile 2  (-0.4-2.1%/year) | Tertile 3  (>2.1%) | p value |
| *Systolic blood pressure* |  |  |  |  | |  |  |  |  |
| Any stroke |  | 971/9958 |  | **1.43 (1.22 – 1.68)** | | **<0.001** | 1 [ref] | **1.23 (1.05 – 1.45)** | **0.01** |
| Ischemic stroke |  | 641/9958 |  | 1.19 (0.98 – 1.45) | | 0.07 | 1 [ref] | 1.07 (0.88 – 1.29) | 0.51 |
| Haemorrhagic stroke |  | 89/9958 |  | 1.42 (0.83 – 2.43) | | 0.20 | 1 [ref] | 1.37 (0.81 – 2.32) | 0.24 |
| Unspecified stroke |  | 241/9958 |  | **2.48 (1.74 – 3.54)** | | **<0.001** | 1 [ref] | **1.91 (1.33 – 2.74)** | **<0.01** |
|  |  |  |  |  | |  |  |  |  |
| *Diastolic blood pressure* | | |  | <-0.8%/year) | |  | (-0.08-2.0%/year) | (>2.0%/year) |  |
| Any stroke |  | 971/9955 |  | **1.30 (1.11 – 1.51)** | | **<0.01** | 1 [ref] | 0.98 (0.83 – 1.15) | 0.79 |
| Ischemic stroke |  | 641/9955 |  | 1.18 (0.98 – 1.43) | | 0.08 | 1 [ref] | 0.88 (0.72 – 1.07) | 0.19 |
| Haemorrhagic stroke |  | 89/9955 |  | 0.83 (0.50 – 1.38) | | 0.48 | 1 [ref] | 0.80 (0.48 – 1.32) | 0.39 |
| Unspecified stroke |  | 241/9955 |  | **2.06 (1.47 – 2.89)** | | **<0.001** | 1 [ref] | **1.51 (1.07 – 2.15)** | **0.02** |

Standard deviation of variance of each tertile for systolic blood pressure: 0.04 (tertile 1), 0.03 (tertile 2), 0.03 (tertile 3). Standard deviation of variance of each tertile for diastolic blood pressure: 0.04 (tertile 1), 0.008 (tertile 2), 0.04 (tertile 3). Abbreviations: n; number of participants with incident stroke, N; total study population, ref; reference.
