# Supplementary material for: Visit-to-visit blood pressure variability and the risk of stroke in the Netherlands: A population-based cohort study
Source: PLoS Med. 2022 Mar 17;19(3):e1003942. doi: 10.1371/journal.pmed.1003942 (PMC8929650; doi:10.1371/journal.pmed.1003942)
Supplement: S8 Table — (DOCX) [file pmed.1003942.s008.docx]

**Table S8**. Association between rise and fall of blood pressure and incident any stroke using different lag periods (unadjusted).

| Lag period (years) |  |  | Any stroke | | | | |
| --- | --- | --- | --- | --- | --- | --- | --- |
|  |  | n/N | Tertile 1  HR (95% CI) | p value | Tertile 2  HR (95% CI) | Tertile 3  HR (95% CI) | p value |
| *Systolic blood pressure* |  |  |  |  |  |  |  |
| 3 |  | 541/7241 | **1.57 (1.36 – 1.78)** | **<0.001** | 1 [ref] | **1.30 (1.08 – 1.51)** | **0.02** |
| 6 |  | 212/4862 | **2.05 (1.67 – 2.44)** | **<0.001** | 1 [ref] | **1.79 (1.41 – 2.16)** | **<0.01** |
| 9 |  | 118/1593 | **2.28 (1.71 – 2.85)** | **<0.01** | 1 [ref] | **1.90 (1.34 – 2.45)** | **0.02** |
|  |  |  |  |  |  |  |  |
| *Diastolic blood pressure* |  |  |  |  |  |  |  |
| 3 |  | 541/7238 | **1.50 (1.03 – 1.97)** | **0.09** | 1 [ref] | 1.14 (0.67 – 1.61) | 0.61 |
| 6 |  | 212/4859 | **1.52 (1.04 – 2.00)** | **0.04** | 1 [ref] | 1.15 (0.67 – 1.64) | 0.57 |
| 9 |  | 118/1591 | 1.46 (0.97 – 1.95) | 0.13 | 1 [ref] | 1.12 (0.62 – 1.62) | 0.67 |

Abbreviations: CI; confidence interval, HR; hazard ratio, n; number of participants with incident any stroke, N; total number of participants at risk, ref; reference.
