# Supplementary material for: Visit-to-visit blood pressure variability and the risk of stroke in the Netherlands: A population-based cohort study
Source: PLoS Med. 2022 Mar 17;19(3):e1003942. doi: 10.1371/journal.pmed.1003942 (PMC8929650; doi:10.1371/journal.pmed.1003942)
Supplement: S9 Table — (DOCX) [file pmed.1003942.s009.docx]

**Table S9**. Association between systolic blood pressure variability and risk of incident any stroke, ischemic stroke, haemorrhagic stroke, and unspecified stroke using complete cases only.

|  |  | n/N |  | Hazard ratio (95% confidence interval) | |
| --- | --- | --- | --- | --- | --- |
|  |  |  |  | per SD | p value |
| *SBP variability* |  |  |  |  |  |
| Any stroke |  | 569/7550 |  | 1.02 (0.91 – 1.13) | 0.78 |
| Ischemic stroke |  | 403/7550 |  | 0.97 (0.85 – 1.12) | 0.71 |
| Haemorrhagic stroke |  | 57/7550 |  | 1.02 (0.69 – 1.50) | 0.93 |
| Unspecified stroke |  | 109/7550 |  | 1.15 (0.92 – 1.42) | 0.22 |

Adjusted for age, sex, mean systolic or diastolic blood pressure, education, body mass index, alcohol consumption, smoking, cholesterol, high-density lipoprotein cholesterol, lipid-lowering medication use, blood pressure-lowering medication use, anti-thrombotic medication use, diabetes mellitus type 2, atrial fibrillation, prior coronary heart disease and blood pressure measuring device. Abbreviations: n; number of participants with incident stroke, N; total number of participants at risk, SBP; systolic blood pressure, SD; standard deviation.
